# Supplementary material for: Demographic isolation and attitudes toward group work in student-selected lab groups
Source: PLoS One. 2024 Sep 24;19(9):e0310918. doi: 10.1371/journal.pone.0310918 (PMC11421786; doi:10.1371/journal.pone.0310918)
Supplement: S1 Table — (DOCX) [file pone.0310918.s001.docx]

**S1 Table. Groupwork Attitude Items**

| **Statements in item “please rate from strongly disagree to strongly agree (5-point scale).”** | **Theoretical Groupwork Factors** |
| --- | --- |
| When I work in a group I do better quality work. | 1. Quality of product and process |
| I enjoy the material more when I work with other students. | 1. Quality of product and process |
| The material is easier to understand when I work with other students. | 1. Quality of product and process |
| My work is better organized when I am in a group. | 1. Quality of product and process |
| The workload is usually less when I work with other students. | 1. Quality of product and process |
| I feel working in groups is a waste of time.* | 1. Quality of product and process |
| My grades improve when I work with other students. | 1. Quality of product and process |
| When I work in a group my work habits improve. | 1. Quality of product and process |
| I become friends with my group members. | 2. Student interdependence |
| I learn to work with students who are different from me. | 2. Student interdependence |
| I help my group members with what I am good at. | 2. Student interdependence |
| It is important to me that my group gets the work done on time. | 2. Student interdependence |
| I also learn when I teach the material to my group members. | 2. Student interdependence |
| Everyone's ideas are needed if we are going to be successful. | 2. Student interdependence |

*Reverse coded in analysis. Items **a**dapted from the SAGE Questionnaire (Kouros et al., 2006).
