# Supplementary material for: Demographic isolation and attitudes toward group work in student-selected lab groups
Source: PLoS One. 2024 Sep 24;19(9):e0310918. doi: 10.1371/journal.pone.0310918 (PMC11421786; doi:10.1371/journal.pone.0310918)
Supplement: S2 Table — (DOCX) [file pone.0310918.s002.docx]

**S2 Table. Multi-level linear regression output for Group Work EAP pretest and posttest scores.**

| **MODEL 1 & 2:** Multi-level linear regression on Pretest and Posttest EAP Scores (n=185) |  |  | **EAP Pretest Score** | | **EAP Posttest Score** | |
| --- | --- | --- | --- | --- | --- | --- |
|  |  |  | **coeff.** | **p-value** | **coeff.** | **p-value** |
|  |  | Pretest Score (EAP) |  | - | 0.689 | **0.000** |
|  | **Fixed Effects** | Female | -0.064 | 0.646 | -0.301 | **0.028** |
|  |  | BHN+ | -0.242 | 0.187 | 0.115 | 0.523 |
|  |  | First-Generation | 0.442 | 0.404 | 0.290 | 0.577 |
|  |  | International | -0.828 | 0.076 | 1.004 | **0.030** |
|  |  | **Isolated** Female | -0.405 | 0.122 | -0.057 | 0.824 |
|  |  | **Isolated** BHN+ | 0.682 | **0.008** | -0.023 | 0.927 |
|  |  | **Isolated** International | -0.030 | 0.957 | -0.155 | 0.775 |
|  |  | **Isolated** First-Generation | 0.533 | 0.370 | -0.494 | 0.397 |
|  |  | Semester | -0.025 | 0.854 | -0.090 | 0.509 |
|  | **Random Effects** | Teaching Assistant | 0.000 |  | 0.000 |  |
|  |  | Group | 0.275 |  | 0.286 |  |
