# Supplementary material for: Demographic isolation and attitudes toward group work in student-selected lab groups
Source: PLoS One. 2024 Sep 24;19(9):e0310918. doi: 10.1371/journal.pone.0310918 (PMC11421786; doi:10.1371/journal.pone.0310918)
Supplement: S3 Table — (DOCX) [file pone.0310918.s003.docx]

**S3 Table. Multi-level linear regression output for Group Work plausible values across five trials to check for accuracy.**

| **MODEL 1:** Multi-level linear regression on PRE plausible values (n=281) |  |  | **PV Trial 1** | | **PV Trial 2** | | **PV Trial 3** | | **PV Trial 4** | | **PV Trial 5** | |
| --- | --- | --- | --- | --- | --- | --- | --- | --- | --- | --- | --- | --- |
|  |  |  | **coeff.** | **p-value** | **coeff.** | **p-value** | **coeff.** | **p-value** | **coeff.** | **p-value** | **coeff.** | **p-value** |
|  | **Fixed Effects** | Female | -0.10 | 0.39 | -0.05 | 0.67 | -0.09 | 0.41 | -0.13 | 0.26 | -0.05 | 0.66 |
|  |  | BHN+ | -0.03 | 0.83 | 0.05 | 0.76 | -0.21 | 0.16 | -0.06 | 0.69 | 0.00 | 0.98 |
|  |  | International | -0.49 | 0.26 | -0.56 | 0.19 | -0.64 | 0.12 | -0.70 | 0.11 | -0.80 | **0.06** |
|  |  | First-Generation | -0.40 | 0.28 | -0.44 | 0.23 | -0.17 | 0.64 | -0.36 | 0.34 | -0.28 | 0.45 |
|  |  | **Isolated** Female | -0.29 | 0.23 | -0.39 | 0.11 | -0.20 | 0.39 | -0.35 | 0.14 | -0.25 | 0.30 |
|  |  | **Isolated** BHN+ | 0.20 | 0.38 | 0.17 | 0.45 | 0.46 | **0.04** | 0.23 | 0.30 | 0.11 | 0.62 |
|  |  | **Isolated** International | 0.15 | 0.76 | 0.42 | 0.39 | 0.41 | 0.38 | 0.53 | 0.29 | 0.69 | 0.16 |
|  |  | **Isolated** First-Generation | 0.54 | 0.18 | 0.63 | 0.11 | 0.44 | 0.25 | 0.45 | 0.26 | 0.32 | 0.42 |
|  |  | Semester | 0.01 | 0.93 | -0.07 | 0.55 | -0.03 | 0.82 | -0.13 | 0.29 | -0.08 | 0.49 |
|  | **Random Effects** | Teaching Assistant | 0.00 |  | 0.00 |  | 0.00 |  | 0.00 |  | 0.00 |  |
|  |  | Group | 0.02 |  | 0.03 |  | 0.00 |  | 0.06 |  | 0.00 |  |
| **MODEL 2:** Multi-level linear regression on POST plausible values controlling for pre and estimating demographic and isolation variable coefficients (n=185) |  |  | **PV Trial 1** | | **PV Trial 2** | | **PV Trial 3** | | **PV Trial 4** | | **PV Trial 5** | |
|  |  |  | coeff. | p-value | coeff. | p-value | coeff. | p-value | coeff. | p-value | coeff. | p-value |
|  | **Fixed Effects** | Pre-Groupwork p.v. | 0.59 | **0.00** | 0.62 | **0.00** | 0.57 | **0.00** | 0.59 | **0.00** | 0.62 | **0.00** |
|  |  | Female | -0.23 | 0.13 | -0.23 | 0.14 | -0.30 | **0.04** | -0.20 | 0.18 | -0.38 | **0.01** |
|  |  | BHN+ | 0.12 | 0.54 | -0.11 | 0.59 | 0.11 | 0.57 | 0.11 | 0.60 | 0.07 | 0.71 |
|  |  | International | 1.01 | **0.04** | 0.92 | **0.08** | 0.97 | **0.04** | 0.79 | 0.12 | 1.00 | **0.05** |
|  |  | First-Generation | 0.25 | 0.66 | 0.20 | 0.74 | 0.22 | 0.69 | 0.26 | 0.65 | 0.69 | 0.22 |
|  |  | **Isolated** Female | -0.32 | 0.26 | -0.07 | 0.82 | -0.08 | 0.78 | -0.03 | 0.92 | -0.11 | 0.70 |
|  |  | **Isolated** BHN+ | 0.16 | 0.58 | 0.01 | 0.97 | -0.08 | 0.78 | 0.10 | 0.72 | 0.00 | 0.99 |
|  |  | **Isolated** International | -0.48 | 0.45 | -0.81 | 0.23 | -0.43 | 0.49 | -0.16 | 0.80 | -0.73 | 0.25 |
|  |  | **Isolated** First-Generation | 0.01 | 0.99 | -0.15 | 0.81 | -0.16 | 0.79 | -0.01 | 0.98 | -0.54 | 0.35 |
|  |  | Semester | -0.17 | 0.26 | -0.06 | 0.72 | -0.07 | 0.65 | -0.07 | 0.65 | -0.13 | 0.38 |
|  | **Random Effects** | Teaching Assistant | 0.00 |  | 0.00 |  | 0.00 |  | 0.00 |  | 0.00 |  |
|  |  | Group | 0.07 |  | 0.05 |  | 0.01 |  | 0.07 |  | 0.13 |  |

Note:  LR tests v. linear: p-value not stat. sig.
