# Supplementary material for: Demographic isolation and attitudes toward group work in student-selected lab groups
Source: PLoS One. 2024 Sep 24;19(9):e0310918. doi: 10.1371/journal.pone.0310918 (PMC11421786; doi:10.1371/journal.pone.0310918)
Supplement: S1 File — (PDF) [file pone.0310918.s004.pdf]

Supporting information

The criteria question: The multiple answer question in the survey asking about the criteria students considered when forming lab group.

Please select all criteria that you used to create or join a research group. Select all that apply.

- I joined the students who were sitting next to me to form a research group.
- I selected my group members based on their GPA and familiarity with the biological concepts.
- I formed a research group with my friends.
- I selected my group members considering their gender identity.
- I selected my group members considering their race/ethnicity.
- I selected my group members based on English being their first language or not.
- I selected my group members considering being first-generation college students or not.
- I selected my group members based on being American or international students.
- I selected my group members considering their age.
- I selected my group members considering their year in college.
- I selected my group members based on the major they are enrolled in.
- I joined the students who were sitting next to me in LECTURE to form a research group

Survey Measures: More details on Groupwork Attitude Items

The fourteen items related to student attitudes that were most relevant to our study are shown in S1 Table. Thematically, eight items related to student attitudes about the quality of their group work product, describing how students feel group work influences their work habits and group accomplishments (e.g., "The material is easier to understand when I work with other students"). Six items were related to student attitudes about interdependence within groups, describing how students believe working together can benefit members of the group (e.g., "Everyone's ideas are needed if we are going to be successful"). We replicated the original 5-point Likert scale from strongly disagree to strongly agree and reverse-coded the negatively worded items as suggested by Kouros and Abrami (2006).

Statistical analyses: More details on item response theory analysis

Given that this study required both the estimation of student scores and the use of those scores as the dependent variable in linear regression, we used item response theory (hereafter IRT) and specifically a partial-credit model to estimate item difficulties and student scores (Wilson, 2023). IRT is particularly well-suited for our analyses given its assumption that students' responses to our items predict their latent knowledge and our estimates inherently include some degree of error. This probabilistic method for estimating student scores can be used to more accurately estimate regression statistics [1-2]. We analyzed two types of student scores, Expected A Posteriori (EAP) scores that average multiple estimates, and plausible values (PV) that include a wider range of possible student scores to account for error more accurately. For each outcome measure, Bayesian partial-credit Rasch models generated 10 PV for each student's group work score and an average of those PV scores which we refer to as the EAP score. While the EAP scores are simpler to interpret, the individual PV scores help to more accurately account for measurement errors and produce the

most accurate regression estimates [3]. In practice, this strategy required multiple iterations of each regression analysis; the first iteration used the EAP scores, and an additional set of five iterations checked those regression coefficients against multiple PVs for consistency (see S2 and S3 Tables).

References

1. Osman TA, Koyuncu İ, Gelbal S. The influence of using plausible values and survey weights on multiple regression and hierarchical linear model parameters. Journal of Measurement and Evaluation in Education and Psychology. 2019;10(3):235-48.
2. Bhaktha N, Lechner CM. To score or not to score? A simulation study on the performance of test scores, plausible values, and SEM, in regression with socio-emotional skill or personality scales as predictors. Frontiers in Psychology. 2021 Oct 15;12:679481.
3. Lechner CM, Bhaktha N, Groskurth K, Bluemke M. Why ability point estimates can be pointless: a primer on using skill measures from large-scale assessments in secondary analyses. Measurement Instruments for the Social Sciences. 2021 Dec;3:1-6
